# 585. Mortality Prediction in Polytrauma Burn Patients: A Bayesian Analysis

**DOI:** 10.1093/jbcr/irag033.358

**Published:** 2026-04-06

**Authors:** Matthew Uzelac, Eloise W Stanton, Clifford C Sheckter

**Affiliations:** Stanford University School of Medicine, Yuba City, CA; Division of Plastic and Reconstructive Surgery, Keck School of Medicine, University of Southern California, Los Angeles, CA; Stanford University School of Medicine, Palo Alto, CA

## Abstract

**Introduction:**

Benchmark burn mortality nomograms such as the revised Baux score exclude patients with concomitant trauma. While polytrauma burn patients represent a minority of all burn admissions, they are the most challenging to treat. An accurate mortality model is needed to facilitate goals-of-care discussions and to set expectations for severely injured polytrauma burn patients.

**Methods:**

The American Burn Association Burn Care Quality Platform (2013-2022; n = 204 678) was utilized to develop a mortality prediction model for polytrauma burn patients. Bayesian regression modeled mortality as a function of known mortality predictors (age, sex, burn total body surface area (TBSA), inhalation injury), traumatic injuries (axial or long bone fractures, hemopneumothorax, solid organ trauma, cardiac arrest, spiral cord lesion, traumatic stroke), and preexisting comorbidities. The model was then updated to include sequela after admission such that clinicians might obtain updated mortality predictions in the event that they develop (acute kidney injury (AKI), acute respiratory failure (ARF), sepsis, pneumonia). The National Trauma Data Bank (2013-2019; n = 51 497) was used to validate the model’s performance.

**Results:**

Concomitant trauma was present in 2.6% of the BCQP cohort (16.9% mortality) and 3.4% of the NTDB cohort (12.9% mortality). Known predictors of burn severity including TBSA (OR 95% CI: 1.09 - 1.09), age (1.06 - 1.07), male sex (0.78 - 0.91), and inhalation injury (4.77 - 5.60) were significantly related to mortality. Of the traumatic injuries, traumatic stroke (3.51 - 7.07), solid organ trauma (1.56 - 4.29), and hemopneumothorax (1.71 - 3.22) had the greatest associations with mortality. Liver disease (4.22 - 6.37), heart failure (1.54 - 2.02), and chronic kidney disease (1.04 - 1.54) were also predictive of death. Using receiver operating characteristic curves, the model achieved an area under the curve of 0.961 with the internal training cohort and 0.980 with the external testing cohort. Its sensitivity ranged from 89-97% and its specificity from 89-91%. Burn sequela were associated with increased mortality, though they did not significantly impact the performance of the model. This might be attributed to the sequela being reflective of the baseline predictors in that patients with AKI or ARF likely sustained larger burns or inhalation injuries, for instance.

**Conclusions:**

This model enables clinicians to provide more accurate survival estimates for polytrauma burn patients, addressing a major limitation of current prognostic tools.

**Applicability of Research to Practice:**

Burn practitioners may use this model to confer expectations of survival and to guide goals-of-care discussions for severely injured polytrauma burn patients.

**Funding for the study:**

N/A.

